# Potential identification of pediatric asthma patients within pediatric research database using low rank matrix decomposition

**DOI:** 10.1186/2043-9113-3-16

**Published:** 2013-09-28

**Authors:** Teeradache Viangteeravat

**Affiliations:** 1 Biomedical Informatics Core, Children’s Foundation Research Institute, Department of Pediatrics, The University of Tennessee Health Science Center, 50 N. Dunlap, 38013, Memphis, TN, USA

**Keywords:** Clinical research, Translational research, Medical informatics, Biomedical informatics, Machine learning, Data mining, Feature extraction, Classification

## Abstract

Asthma is a prevalent disease in pediatric patients and most of the cases begin at very early years of life in children. Early identification of patients at high risk of developing the disease can alert us to provide them the best treatment to manage asthma symptoms. Often evaluating patients with high risk of developing asthma from huge data sets (e.g., electronic medical record) is challenging and very time consuming, and lack of complex analysis of data or proper clinical logic determination might produce invalid results and irrelevant treatments. In this article, we used data from the Pediatric Research Database (PRD) to develop an asthma prediction model from past All Patient Refined Diagnosis Related Groupings (APR-DRGs) coding assignments. The knowledge gleamed in this asthma prediction model, from both routinely use by physicians and experimental findings, will become fused into a knowledge-based database for dissemination to those involved with asthma patients. Success with this model may lead to expansion with other diseases.

## Background

### Data mining in medical informatics

Because of their predictive power, various healthcare systems are attempting to use available data mining techniques to discover hidden relationships as well as trends in huge data available within the clinical database and convert it to valuable information that can be used by physicians and other clinical decision markers. In general, data mining techniques can learn from what was happened in past examples and model oftentimes non-linear relationships between independent and dependent variables. The resulting model provides formalized knowledge and prediction of outcome. For example, Shekar et al. used data mining based decision tree algorithm to discover the most common refractive error in both male and female [1]. Palaniappan et al. presented a prototype that combines the strengths of both an online analytical processing (OLAP) and data mining techniques for clinical decision support systems (DSS) [2]. Jonathan et al. used data mining techniques to explore the factors contributing to cost of prenatal care and outcomes [3]. Chae et al. used data mining approach analysis in health insurance domain [4]. With advanced data mining techniques to help evaluate healthcare utilization costs for employees and dependents in organizations [5].

More advanced machine learning methods, such as artificial neural networks and support vector machines, have been adopted to use in various areas of biomedical and bioinformatics, including genomics and proteomics [6]. For biological data, clustering is probably the most widely used data mining technique, such as clustering analysis, hierarchical clustering, *k*-means clustering, backpropagation neural networks, self-organization maps, fuzzy clustering, expectation maximization, and support vector machines [7,8]. Bayesian models were widely used to classify data into predefined classes based on a set of features. Given the training examples, a Bayesian model stores the probability of each class, the probability of each feature, and the probability of each feature given each class. When a new unseen example occurred, it can be classified according to these probabilities [9,10]. This classification technique is one of the most widely used in medical data mining. Decision tree models, such as the Iterative Dichotomiser 3 (ID3) Heuristic techniques belong to the subfield of machine learning. The ID3 Heuristic uses a technique called "entropy" to measure disorder in a set of data [11,12]. The idea behind the ID3 Heuristic is to find the best attribute to classify the records in the data set. The outcome is learned rules and a model used to predict unseen examples based on past seen examples. Non-negative matrix factorization (NMF) has been widely used in the field of text mining applications [13,14]. The only constraint that is unique from other methods is factorization of two matrices *W* and *H* from *V* (i.e., *nmf* (*V*) *→ WH*) must be non-negative or all elements must be equal to or greater than zero. Typically, *W* and *H* are initialized with random non-negative values to start the NMF algorithm. The convergent time is varied and local minimum is not guaranteed [15].

Here, we are working on a methodology and classification technique in data mining called Low Rank Matrix Decomposition (LRMD) to allow computer to learn from what has happened in the past APR-DRGs datasets for asthma, able to extract dominant features, and then predict outcomes. The summary of APR-DRGs and the mathematics behind LRMD is discussed further below.

### All patient refined diagnosis related groups (APR-DRGs)

APR-DRG is a grouping methodology developed in a joint effort between 3M Health Information Systems (HIS) and National Association of Children’s Hospitals and Related Institutions (NACHRI). APR-DRGs are proprietary and have the most comprehensive and complete classification of any severity of illness system for pediatric patients. It was designed to be more appropriate for general population patients than the old Diagnosis Related Group (DRG) [16]. While the DRG was designed and normed on Medicare patients only, the APR-DRG was designed and normed on a general population. We use APR-DRG based weights normed on a pediatric patient population. There are 316 APR-DRGs, such common APR-DRG codes include but not limited to 138 Bronchiolitis/RSV pneumonia, 141 Asthma, 160 Major repair of heart anomaly, 225 Appendectomy, 420 Diabetes, 440 Kidney transplant, 662 Sickle cell anemia crisis, and 758 Childhood behavioral disorder. Each group has 4 severity levels of illnesses (SOI) and 4 risk levels of mortality (ROM) while the DRG and Medicare Service – Diseases Related Groups (MS-DRG) have only a single severity and risk of mortality per group. For example, there are multiple diagnosis codes for asthma and an encounter might have asthma as principal diagnosis or a secondary diagnosis and if the encounter was primarily for asthma treatment, then the APR-DRG code will be 141 and all asthma encounters will be assigned the same APR-DRG code. In our internal system we code inpatient encounters to APR-DRG as well as DRG. We have available from our PRD back through 2009 [17], including Emergency Room (ER), Ambulatory Surgery (AS), and Observation (OBS) encounters.

## Methods

### Singular value decomposition

In general, the Singular Value Decomposition method is a method for decomposition of any matrix A∈R^MxN^ where M ≥ N in a product of UV^T^ , where U∈R^Mxk^ and V∈R^Nxk^[18,19]. Since any rank *k* matrix can be decomposed in such a way, and any pair of such matrices yields a rank *k* matrix, the problem becomes as an unconstrained minimization over pairs of matrices (U ,V ) with the minimization objective

$$f\left(U,V\right)=\text{min}{\left||A-A^{\left(k\right)}|\right|}_{2}^{2}$$

(1) $$=\text{min}(\sum {}_{n=1}^{N}\sum {}_{m=1}^{M}\;|A\left(m,n\right)-A^{\left(k\right)}\left(m,n\right){|}^{2})$$

$$=\text{min}{\left||A-U^{\left(k\right)}V^{{\left(k\right)}^{T}}|\right|}_{2}^{2}$$

$$=\text{min}\left\{{\left[A-U^{\left(k\right)}V^{{\left(k\right)}^{T}}\right]}^{T}\left[A-U^{\left(k\right)}V^{{\left(k\right)}^{T}}\right]\right\}$$

Where *A*^(*k*)^ is a rank *k* approximation of matrix A. To find the optimum choices of U,V in *l*_2_ norm sense [20,21], the partial derivatives of the objective *f* (*U,V*) with respect to U,V are

(2) $$\frac{\partial f\left(U,V\right)}{\partial U}=2\left(UV^{T}-A\right)V$$

(3) $$\frac{\partial f\left(U,V\right)}{\partial V}=2\left(VU^{T}-A^{T}\right)U$$

Solving $\frac{\partial f\left(U,V\right)}{\partial U}=0$ for U yields *U* = *AV*(*V*^*T*^*V*)^- 1^. By considering an orthogonal solution, then U = Λ is diagonal such that U = AV. Substituting back into $\frac{\partial f\left(U,V\right)}{\partial V}=0$, we have

(4) $$VU^{T}U-A^{T}U=V\Lambda -A^{T}\mathrm{AV}=0$$

The columns of V are mapped by *A*^*T*^*A* to multiples of themselves, i.e., they are eigenvectors of *A*^*T*^*A*. Therefore, the gradient $\frac{\partial f\left(U,V\right)}{\partial \left(U,V\right)}$ vanishes at an orthogonal (U,V) if and only if the columns of V are eigenvectors of *A*^*T*^*A* and the column of U are eigenvectors of *AA*^*T*^, scaled by the square root of their eigenvalues [18,19]. More generally, the gradient vanishes at any (U,V) if and only if the columns of U are spanned by eigenvector of *AA*^*T*^ and the columns of V are spanned by eigenvector of *A*^*T*^*A*. In term of the singular value decomposition, $A=U_{o}SV_{o}^{T}$ the gradient vanishes at (U,V) if and only if there exist matrices $P_{U}^{T}P_{V}=I\in R^{\mathrm{kxk}}$ such that *U* = *U*_*O*_ *SP*_*U*_ and *V* = *V*_*O*_ *P*_*V*_. Thus, using singular eigenvectors that corresponds to the largest singular values can represent the global properties (i.e., feature vectors) of A with satisfying the minimization under *l*_2_ norm sense [19].

### Low rank matrix decomposition

Suppose that it is desired to represent matrix *X*∈*R*^*MxN*^ as a sum of simple rank one matrices so as to capture the nature of the matrix in which matrix *X* is to be represented by the summation of *r,* i.e., rank of matrix. In this case, the outer products can be written as:

(5) $$X=\sum {}_{i=1}^{r}u_{i}v_{i}^{T}$$

Where *X* ∈ *R*^*M x N*^, {*u*_1_, *u*_2_, …, *u*_*r*_} and {*v*_1_, *v*_2_, …, *v*_*r*_} vectors each represents linearly independent column vectors with dimensions M and N, respectively. The constituent outer product $u_{i}v_{i}^{T}$ is rank one in which the MxN matrix whose column (row) vectors are each a linear multiple of vector *u*_*i*_*(v*_*i*_*)*. To be more precise, a necessary condition is that the vector set {*u*_*1*_*, u*_*2*_*, …, u*_*r*_} must form a basis for the column space of matrix X and the vector set $\left\{v_{1}^{T},v_{2}^{T},\ldots ,v_{r}^{T}\right\}$ should form a basis for the row space of matrix X. It is noted, however, that there will exist an infinite number of distinct selections of these basis vectors for the case r ≥ 2. It then follows that there will be an infinite number of distinct ranks when the decomposition of a matrix has rank r ≥ 2. The ultimate selection to be made is typically based on the application as well as computational considerations. To provide a mathematically based method for selecting the required basis vectors, let us consider the functional relationship

(6) $$f_{k}\left(\left\{u_{i}\right\},\left\{v_{i}\right\}\right)={\left||X-\sum {}_{i=1}^{k}u_{i}v_{i}^{T}|\right|}_{p}$$

For 1 ≤ *k* ≤ *r* and *p* = 1,2 where the integer *k* ranges in the interval 1 ≤ *k* ≤ *r*.

It can be readily shown that the function (6) represents a convex function of the set {*u*_*i*_} for a fixed set of {*v*_*i*_} and vice-versa. For the proof, please refer to [22]. The convexity property is important since it ensures that any local minimum of *f*_*k*_ (*v*) (i.e., *u* is fixed) and vice-versa is also a global minimum. With regard to the above equation, a specific selection of the vector sets {*u*_1_,*u*_2_,…,*u*_*k*_}∈ *R*^*M*^ and {*v*_1_,*v*_2_,…,*v*_*k*_}∈ *R*^*N*^ is to be made so as to minimize this functional. The optimal selection will then provide the best rank *k* approximation of matrix *X* in the *lp* norm sense, as designated by

(7) $$X^{\left(k\right)}=\sum {}_{i=1}^{k}u_{i}^{o}v_{i}^{o^{T}}$$

This optimal matrix is said to be the best rank *k* approximation of matrix *X* in the *lp* norm sense. For convenience, we express equation (5) in a normalized form as:

(8) $$X^{\left(k\right)}=\sum {}_{i=1}^{k}\sigma_{i}^{o}u_{i}^{o}v_{i}^{o^{T}}$$

Where ${||u}_{i}^{o}{\left|\right|}_{p}={||v}_{i}^{o}{\left|\right|}_{p}=1$ and σ_*i*_^*o*^ are positive scalars. The most employed matrix decomposition procedure is the Singular Value Decomposition (SVD). The SVD method provides an effective method for mitigating the deleterious effects of additive noise and is characterized by the function *f*_*k*_({*u*_*i*_},{*v*_*i*_}) in the *l*_2_ norm sense, that is

(9) $${\left||X-X^{\left(k\right)}|\right|}_{2}=\sqrt{\sum {}_{n=1}^{N}\sum {}_{m=1}^{M}|X\left(m,n\right)-X^{\left(k\right)}\left(m,n\right){|}^{2}}$$

The use of the *l*_1_ norm criterion can be of practical use when analyzing data that contains data outliers. Namely, it would be useful to express this equation (9) as an objective function that optimizes the best rank *k* approximation of matrix *X*∈*R*^*MxN*^ as measured for the case of the *l*_1_ norm criterion. That is

(10) $${\left||X-X^{\left(k\right)}|\right|}_{1}=\sum {}_{n=1}^{N}\sum {}_{m=1}^{M}|X\left(m,n\right)-X^{\left(k\right)}\left(m,n\right)|$$

In order to attempt to find the optimum solution which minimizes the objective function (10), we introduce a concept, called Alternating Optimization, This optimization concept is explained a detailed below.

### Alternating optimization

We can rewrite the equation (10) in term of matrices U and V as *f* (*U*, *V*) = ||*X* - *UV*^*T*^||_1_ by fixing U, then objective function becomes:

(11) $$f\left(V\right)={\left||X-U_{\mathrm{fix}}V^{T}|\right|}_{1}$$

Where $X=\left[{\vec{x}}_{{}_{1}}\;{\vec{x}}_{{}_{2}}.\;.\;.\;.\;.{\vec{x}}_{{}_{n}}\right],V=\left[{\vec{v}}_{{}_{1}}\;{\vec{v}}_{{}_{2}}.....{\vec{v}}_{{}_{k}}\right]$ and similarly the column of *V*^*T*^ are denoted by $V^{T}=\left[{\tilde{v}}_{1}\;{\tilde{v}}_{2}.\;.\;.\;.\;.{\tilde{v}}_{n}\right]$. It is straightforward to see that f (V) can be rewritten as a sum of independent criteria

(12) $$f\left(V\right)=\sum_{i=l}^{n}║{\vec{x}}_{{}_{1}})-U_{\mathrm{fix}}{\tilde{v}}_{{}_{i}}║{}_{1})$$

where each $||{\tilde{x}}_{i}-U_{\mathrm{fix}}{\tilde{v}}_{i}{\left|\right|}_{1}$ term may be minimized independently by selecting an appropriate. The solution method for each of these subproblems is given in [23]. Grouping the resulting ${\tilde{v}}_{i}$ together to obtain *V*^*T*^, we get a solution for equation (11). On the other hand, by fixing V, the objective function can be expressed as:

(13) $$\begin{array}{ll} \;f\left(U\right) & =||X-UV_{\mathrm{fix}}^{T}{\left|\right|}_{1}\\ & =||X^{T}-V_{\mathrm{fix}}U^{T}{\left|\right|}_{1} \end{array}$$

And a similar method may be used to solve for U. The iteration process proceeded by finding ${\tilde{v}}_{i}$ and then finding ${\tilde{u}}_{i}$ (i.e., the alternating optimization) is continued until a stopping criterion is met (i.e., the matrix from two successive iterations are sufficiently close). For example, $||X_{i-1}^{\left(k\right)}-X_{i}^{\left(k\right)}{\left|\right|}_{2}<\epsilon ,\epsilon =10^{-7}$. However, it must be noted that finding a global minimum is not guaranteed. In the following section, we establish a guideline for selection of the stopping criteria.

### Selection criterion

In this section, let us direct our attention to the selection criteria for the initial choice for *U*, where *U*∈*R*^*Mxk*^. We note that for the following cases where (i) rank *k* = 1 approximation and (ii) rank 1 < *k ≤ r*, then *r =* rank (*X*). In order to take the global data into account, a good choice of initial value of *U* for a rank *k* = 1 (i.e., *U*∈*R*^*Mx*1^) approximation may be obtained as follows. First, we compute the *l*_1_ norm of each column vector in *X*, and denoted this norm by $x_{1}^{c},x_{2}^{c},\ldots ,x_{n}^{c}$. Next compute the *l*_1_norm of each row vector in *X*, and denoted this norm by $x_{1}^{r},x_{2}^{r},\ldots ,x_{m}^{r}$. Now we find the maximum value in $\left\{x_{1}^{c},x_{2}^{c},x_{n}^{c},x_{1}^{r},x_{2}^{r},\ldots ,x_{m}^{r}\right\}$. If the maximum corresponds to a column norm, say from column j, then chose that column (i.e., *U* = *X* (:,*j* )) as the initial choice for *U*. If the maximum corresponds to a row norm, say row I, then we start with the transposed form of the criterion in (11) and we chose that row (i.e., *V*^T^ = *X* (*i*,:)) as the initial choice for *V*^*T*^. We can also extend the previous concept to find the initial choice for *U* for the rank *k* = 2. Essentially, we apply the rank one approximation twice in succession. Therefore our objective function can be expressed as:

(14) $$\begin{array}{ll} \text{min}||E_{2}{\left|\right|}_{1} & =\underset{u_{1},u_{2},v_{1},v_{2}}{\underset{︸}{\text{min}}}||X-\left[u_{1}\;u_{2}\right]{\left[v_{1\;}v_{2}\right]}^{T}{\left|\right|}_{1}\\ & =\underset{U,V}{\underset{︸}{\text{min}}}||X-UV^{T}{\left|\right|}_{1} \end{array}$$

Where *U* = [*u*_1_*u*_2_] and *V* = [*v*_1_*v*_2_], *u*_1_,*u*_2_,*v*_1_,*v*_2_ are vectors. Therefore the initial choice for U (rank *k =* 2) is *U* = [*u*_1_*u*_2_] (i.e., two largest *l*_1_ column or row norm). In a similar fashion, a selection criterion for the initial for U for rank *k* (1 *< k ≤ r* ) can be also obtained. Thus the column space of *X* (i.e., U) represents a feature vector that is considered as a global property (i.e., the best low rank approximation) of *X* that minimizes the above objective function under *l*_1_ norm sense [22].

### Convergence subsequence

The error sequence happened in each iteration can be expressed as:

(15) $$E_{i}\left(U,V\right)=||X-X_{i}^{\left(k\right)}{\left|\right|}_{p}\;\mathrm{where}\;X_{i}{}^{\left(k\right)}=U_{i}V_{i}^{T}\;\mathrm{and}\;p=1,2$$

Since the error sequence is bounded below (i.e., *E*(*U,V*) ≥0)) we have

(16) $$E_{i}\left(U,V\right)=||X-X_{i}^{\left(k\right)}{\left|\right|}_{p}\leq E_{1}\;\mathrm{where}\;E_{1}\geq 0$$

And lim _*i* → *∞*_*E*_*i*_ = *E*_*final*_ ≥ 0. Therefore the entire infinite length sequence lies inside a hypersphere (i.e., a closed and bounded set of points) of finite volume centered at *X* and with a radius of *E*_1_. Since this hypersphere has finite volume, it is possible to construct a finite number of smaller hypersphere, each with radius ϵ > 0, such that the union of all these small hyperspheres contains the large hypersphere of radius *E*_1_. For all ϵ > 0 there will be at least one hypersphere of radius ϵ containing an infinite number of points of the sequence. Thus, there is at least one cluster point. The cluster point is the limit of a convergent subsequence. Therefore, we know that the sequence of *X*_*i*_^(k)^, produced by the algorithm must contain at least one convergent subsequence.

### Feature extraction methodology

**Figure 1:**
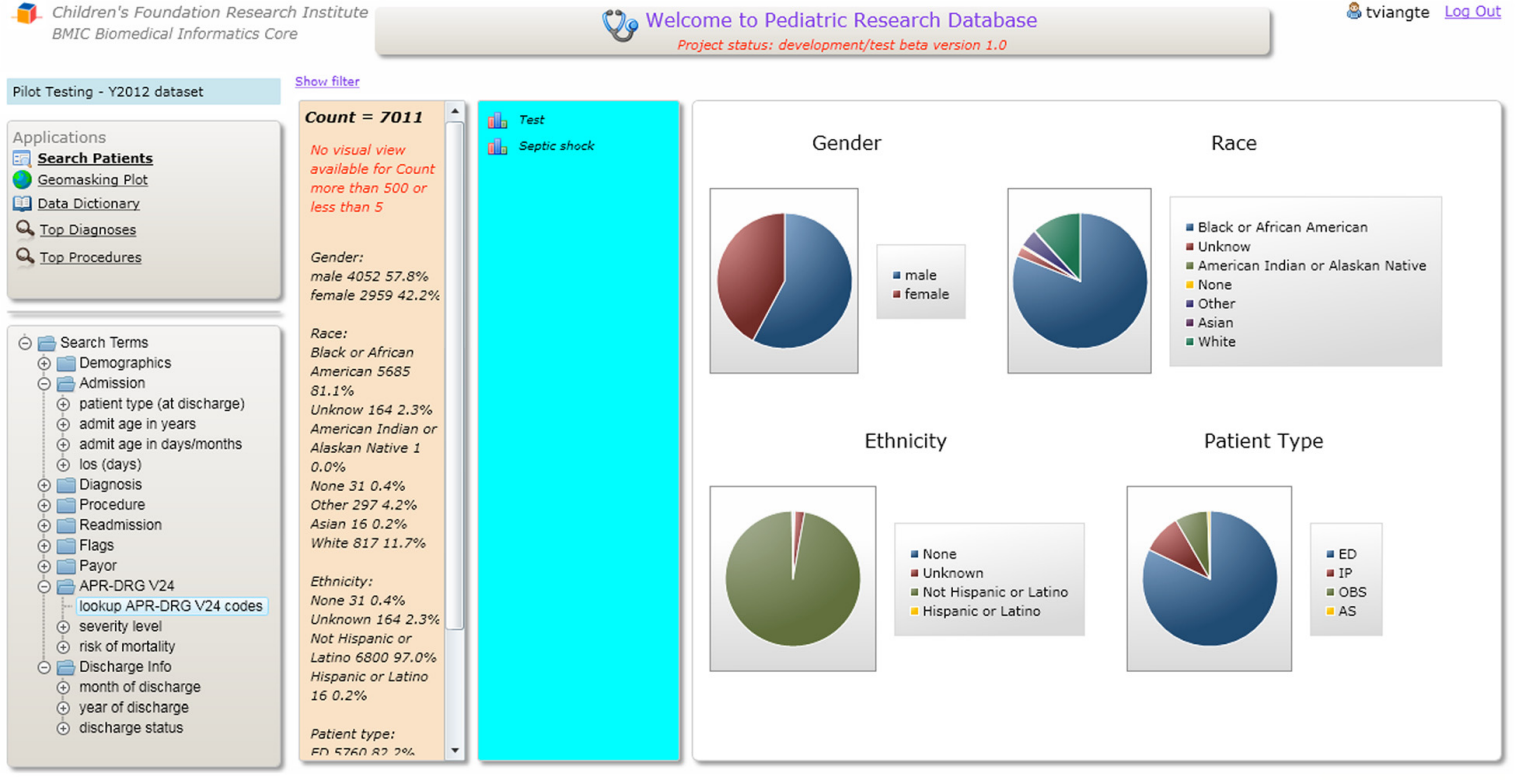
The PRD search cohort demonstrates the ability to search for possible asthma cases based on APR-DRGs.

**Figure 2:**
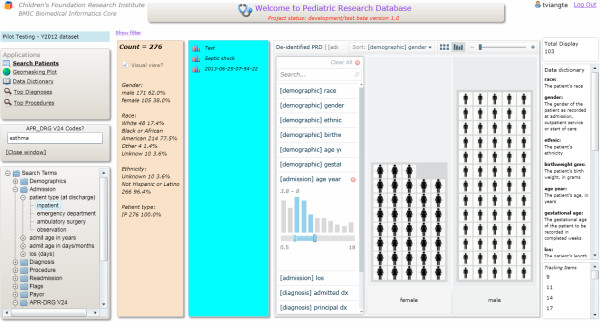
The PRD visual analytic feature demonstrates the ability to sort and filter by various criteria (tree view sorted by patient’s gender and filtered by patient’s age).

For the purpose of this preliminary study, we acquired de-identified data sets from PRD that demonstrate patient visits in year 2012. The total number of observations includes 92,175 encounters. Among all encounters, we selected encounters that have APR-DRG code = 141 Asthma, 144 Respiratory signs & minor diagnoses, 131 Cystic fibrosis – pulmonary disease, and 132 BPD & chronic respiratory for our initial datasets. The total number of meeting criteria is 8,895 encounters for 7,011 distinct patient records (see Figure 1 and Figure 2). Among all patients, 57.8% (4,052) were male, 11.7% (817) were white, and 81.1% (5,685) were black or African-American. The PRD has the UTHSC Institutional Review Board (IRB) approval for the creation and maintenance of the database. The waiver applies to the medical records of patients who received care in 2009 or later.

The text parsing software and natural language toolkit [24] (written in Python) were used to parse all encounter data sets for this preliminary study. If *X =* [*x*_ij_] defines the *m* × *n* term-by-encounter matrix for decomposition. Each element or component *x*_ij_ of the matrix *X* defines a weighted frequency at which term *i* occurs in encounter *j*, where term *i*∈ {gender, age, discharge status, admitting diagnosis, secondary diagnoses, principal diagnosis, principal procedure, secondary procedures}. The corpus stop words from NLTK were used to filter out unimportant terms.

**Figure 3:**
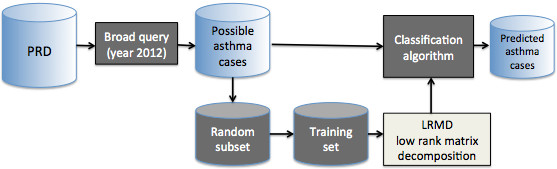
Classification workflow.

In evaluating the classification performance, we randomly selected subset 1,200 encounters and divided into a number of four subsets of equal size (i.e., four-fold cross validation). The system is trained and tested for four iterations (see Figure 3).

In each iteration, three subsets of data are used as training data and the remaining set is used as testing data. In rotation, each subset of data serves as the testing set in exactly one iteration. The rank *U* used to test the LRMD was *k* = 4. Hence, the *U* and *V* matrix factors were number of terms × 4 and 4 × 1200, respectively. The percentage of possible asthma encounters used for training in our testing was 900 encounters and the remaining 300 encounters were used for testing our classifier. The initial matrix factors *U* and *V* were selected to meet our Selection criterion (see Selection Criterion) and alternating iteration was continued until the matrix from two successive iterations are sufficiently close (see Alternating optimization). All classification results were obtained using Python version 2.7.4.

## Results

Table 1 demonstrates an example of dominant features for the classifier, when applied to training data sets (randomly selected 900 out of a 1,200 encounters). We note that among all features, admitting diagnosis = 786.07 (wheezing), secondary diagnosis = 786.05 (shortness of breath), age 4–8, and having family history of asthma (ICD-9-CM = v175) would potentially progress toward asthma, i.e., APR-DRG code = 141 asthma. The 2nd Feature shows asthma patients with pneumonia condition (ICD-9-CM = 486.00) during the length of stay in a hospital. The 4th Feature demonstrates the connection between asthma symptoms and another pulmonary condition known as bronchitis symptom (ICD-9-CM = 466.0). When the two conditions co-exist, bronchitis can cause patients with asthma to make their asthma symptoms worse, i.e., an asthma attack.

**Table 1:** Example of dominant features using LRMD

**Variables**	**1**^**st **^**Feature**	**2**^**nd **^**Feature**	**3**^**rd **^**Feature**	**4**^**th **^**Feature**	**5**^**th **^**Feature**
admitting diagnoses (ICD-9-CM)	786.07	786.07	786.07	786.07	786.07
	493.90	786.09	493.92		493.92
secondary diagnoses (ICD-9-CM)	v175	486.00	786.05	786.05	v175
	530.81	692.9	v175	780.60	692.9
	786.05	v175	785.0	692.9	785.0
			v174.9	786.2	787.03
				466.0	
				v175	
principal diagnoses (ICD-9-CM)	493.92	493.92	493.92	493.92	493.92
	494.90	493.90	786.06	493.91	
	493.91				
principal procedures (ICD-9-CM)	N/A	939.4	N/A	N/A	939.4
age (year)	4-7	3.5-7	4-6.5	4-6	4.5-8
gender	male	female	female	female	male
discharge status	home	home	home	home	home

**Figure 4:**
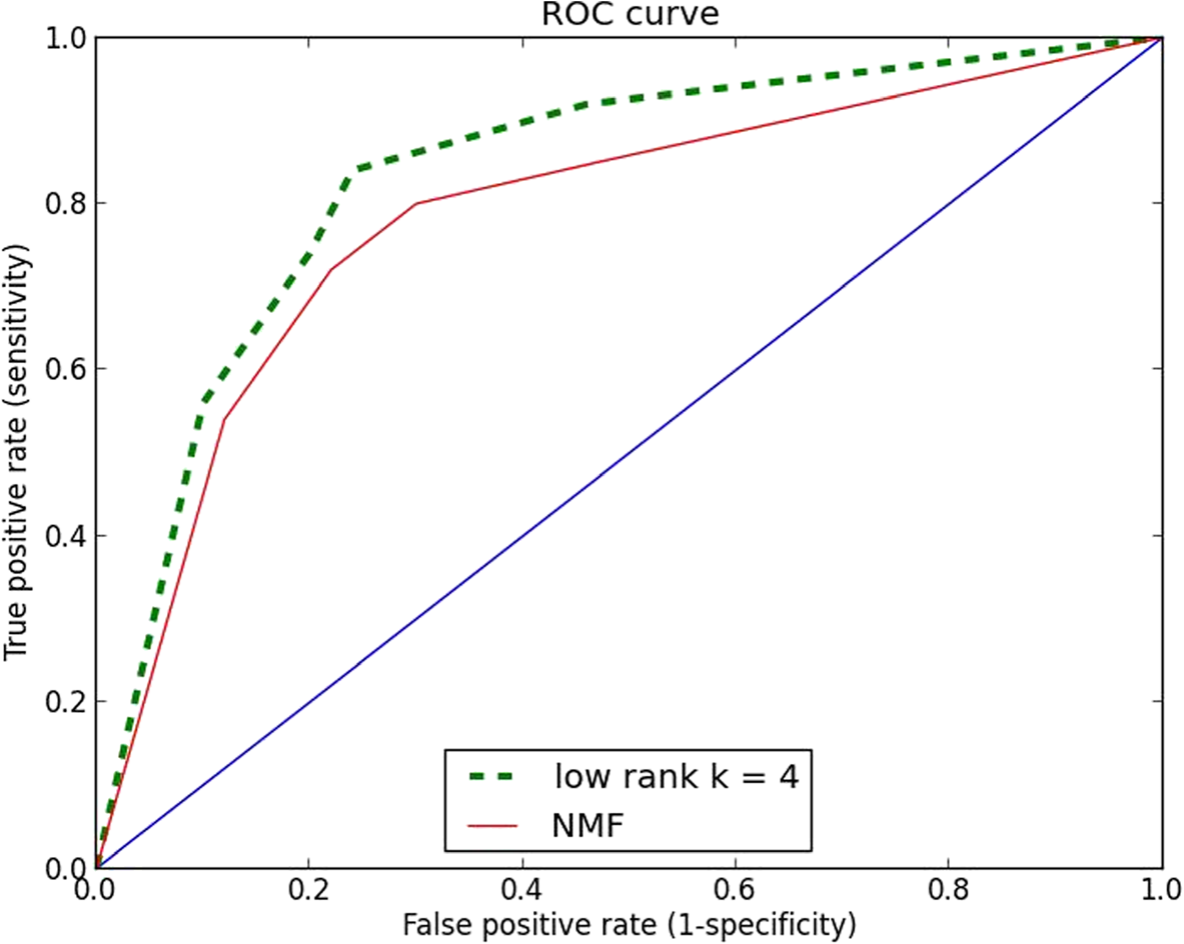
The receiver operating characteristic (ROC) shows sensitivity and specificity of our classifier.

To evaluate the performance of our classifier for this preliminary study, we plot a receiver operating characteristic (ROC). Figure 4 shows the receiver operating characteristic (ROC) curves (true positive rate versus false positive rate).

Our goal is to maximize the number of true positives (correctly diagnosed asthmas) with an acceptable number of false positives (false alarm). From Table 2 and Figure 4, we note that as sensitivity goes up, a specificity goes down. We can begin to see the trade-offs when we insist an higher sensitivity (fewer missed asthmas) the result is lower specificity, and more false positive. In practice, it is much worse to miss a asthma than to endure unnecessary treatment, so we tend to choose a higher sensitivity cut off (e.g., cutoff score > 0.65 or > 0.75). As it is apparent from Table 2, LRMD yields very promising results for disease identification and classification. However, we still have much work to do to enhance the LRMD classifier and it is discussed further below.

**Table 2:** Sensitivity and specificity

**Cutoff score for similarity to features in training set (1 = perfect correlation and 0 = no correlation)**	**LRMD**		**NMF**	
	**Sensitivity**	**Specificity**	**Sensitivity**	**Specificity**
> 0.65	0.92	0.54	0.85	0.53
> 0.75	0.84	0.76	0.8	0.7
> 0.85	0.74	0.8	0.72	0.78
> 0.9	0.56	0.9	0.54	0.88

## Discussion

The results presented in this paper should not be taken as an accurate representation of our patient data (as it does not include all the data records). These data are meant to demonstrate the potential of PRD and the feasibility of data mining technique using LRMD. Additional experiments with a larger number of features (rank *k* > 4) and encounter data sets (2009 – 2012) should produce better models to capture the diversity of contexts described by those encounters. Using ICD-9-CM has limitations because they are generally used for billing purposes and not for clinical research. We are planning to access free-text fields in the near future, such as physician and clinician notes, and include them into our classifier. Additional socio-demographic variables such as incomes, type of insurance, environment, nutrition, genome and comorbidity covariants could potentially be added to the model to support the evaluation of potential causes for readmission.

## Conclusions

Using data mining technique to learn from past examples within rich data sources such as electronic medical records not only permits users to detect expected events, such as might be predicted by models, but also helps users discover the unexpected patterns and relationships that can then be examined and assessed to develop new insights. We hope that learned rules from the LRMD technique will greatly advance progress toward the goal of identifying high risk of pediatric asthma patient and help support clinical decisions.

## Competing interests

The author declares that they have no competing interests.
